# Protein-Decorated Microbubbles for Ultrasound-Mediated Cell Surface Manipulation

**DOI:** 10.1021/acsabm.3c00861

**Published:** 2023-12-04

**Authors:** Veerle A. Brans, Michael D. Gray, Erdinc Sezgin, Eleanor P. J. Stride

**Affiliations:** † Department of Engineering Science, Institute of Biomedical Engineering, University of Oxford, Oxford OX3 7DL, U.K.; ‡ Science for Life Laboratory, Department of Women’s and Children’s Health, Karolinska Institutet, 17165 Solna, Sweden

**Keywords:** microbubbles, ultrasound, recombinant protein, metallochelation, microbubble−cell interactions, cell membrane tagging, confocal microscopy

## Abstract

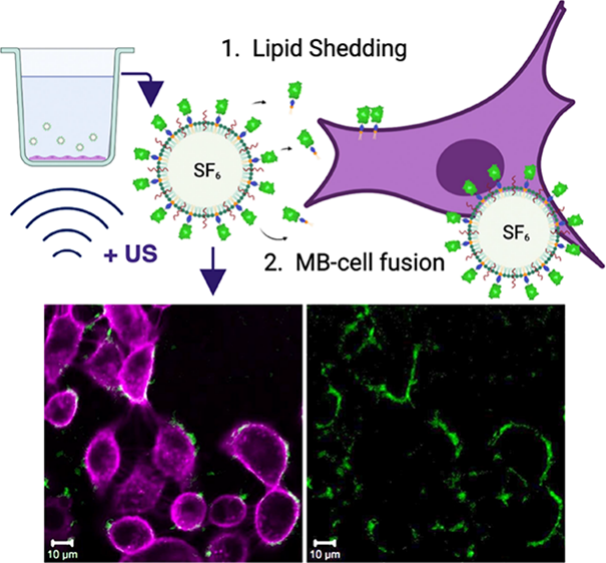

Delivering cargo to the cell membranes of specific cell types in the body is a major challenge for a range of treatments, including immunotherapy. This study investigates employing protein-decorated microbubbles (MBs) and ultrasound (US) to “tag” cellular membranes of interest with a specific protein. Phospholipid-coated MBs were produced and functionalized with a model protein using a metallochelating complex through an NTA(Ni) and histidine residue interaction. Successful “tagging” of the cellular membrane was observed using microscopy in adherent cells and was promoted by US exposure. Further modification of the MB surface to enable selective binding to target cells was then achieved by functionalizing the MBs with a targeting protein (transferrin) that specifically binds to a receptor on the target cell membrane. Attachment and subsequent transfer of material from MBs functionalized with transferrin to the target cells significantly increased, even in the absence of US. This work demonstrates the potential of these MBs as a platform for the noninvasive delivery of proteins to the surface of specific cell types.

## 1 Introduction

Cell-specific delivery of drugs or biomolecules in the human body continues to be a major challenge in biomedicine. For example, one immunotherapy strategy^1^ involves removing T cells from patients and genetically altering them so that they display chimeric antigen receptors (CARs) on the membrane—proteins capable of recognizing cancer cells as well as activating the T cell itself. However, these CAR-T cell therapies are expensive, risky, and technically demanding. This is partly because this type of adaptive therapy requires complex *ex vivo* procedures. A method to overcome this bottleneck would be highly beneficial for targeted therapies, and there is an increasing interest in the development of therapies capable of modulating the immune system in vivo, including biomaterials-based strategies.^2^ This work demonstrates a potential method to manipulate the cell surface using microbubbles (MBs) and ultrasound (US).

In recent years, the use of US has been explored in a therapeutic context for applications such as thrombolysis,^3^ blood–brain barrier opening,^4^ and gene and drug delivery.^5^ The key phenomenon underpinning the therapeutic potential of US is cavitation: the nucleation, growth, and oscillation of gaseous cavities due to changes in fluid pressure.^6^ Cavitation results in a myriad of mechanical, thermal, and chemical mechanisms underpinning biological effects such as shear-induced permeabilization,^7^ high-velocity microjets,^8^ heating,^9^ and the generation of highly reactive species and electromagnetic radiation.^10^ The presence of exogenous or artificial cavitation nuclei substantially reduces the input energy required to produce this broad range of effects. The most commonly used nuclei are gas MBs, which consist of a high molecular weight gaseous core and a lipid shell and are typically 1–2 μm in diameter.^11^ However, the use of MBs and US focuses on two main avenues for drug delivery and intracellular uptake: (i) via temporary membrane pores, known as sonoporation,^12,13^ and (ii) enhanced endocytosis.^14^ In 2016, De Cock et al. proposed a third application that relies on MB–cell interactions, so-called sonoprinting.^15^ Sonoprinting is the direct deposition of nanoparticles, loaded on MBs, in patches onto the cell membrane following US exposure. Furthermore, various studies have demonstrated evidence for the transfer of MB cargo and lipids, as well as MB–cell fusion through exchanging or mixing of lipids, including as a function of US exposure during which “shedding” of lipid from the MB surface occurs.^15−17^

Recent studies have unveiled important functions of plasma membrane lipid and cell surface signaling molecule dynamics in regulating T cell signaling, and, hence, modulation of these membrane lipids can be exploited to harness T cell activity.^18,19^ Therefore, there is a need for an MB formulation that, instead of delivering lipids or other cargo intracellularly, “tags”, like sonoprinting,^15,16^ the cell membrane with relevant proteins to increase either the visibility of target cells to the immune system or the potency of immune cells to eliminate diseased cells.

In 2018, Jenkins et al. explored the use of giant unilamellar vesicles (GUVs), as opposed to MBs, to study the interactions of live T cells, B cells, and mast cells with signaling proteins normally present at immune cell–cell contacts and observed dynamic spatiotemporal regulation of signaling proteins, including kinases, responsible for immune activation.^20^ To functionalize the GUVs with signaling proteins, Jenkins et al. incorporated nickel-nitrilotriacetic acid (Ni-NTA)-functionalized lipids into these GUVs, which are capable of binding directly to His-tagged surface proteins.

Although studying live immune cells interacting with free-standing vesicles offers a way to elucidate passive and active processes involved in the immune response, these systems are limited in their use: vesicles cannot be used as drug carriers due to their inability to fuse with the cell membrane, and endocytosis reduces their delivery efficiency. Encapsulated MBs, on the other hand, are known to fuse with the cell membrane and can, therefore, potentially be used as efficient drug carriers.^21,22^

In 2011, Lukáč et al. first showed the construction of what they referred to as surface-modified metallochelating MBs, or, in other words, MBs with an NTA(Ni)-functionalized lipid incorporated in the shell, as an imaging contrast agent.^23^ They argued that the reversible bond, fast binding kinetics at room temperature, high affinity, and low if any immunogenicity made the Ni-His binding method a good candidate for use in MBs for in vivo drug delivery. Prior to their work, others used this binding method albeit in different systems and applications.^24−26^

Here, we report on the feasibility of designing an MB agent functionalized with a protein to tag target cell membranes. First, we characterize nickelated MBs functionalized with a His-tagged green fluorescent protein (His-GFP) as a model protein. This includes an assessment of the MB population statistics, the fluorescence properties, and the stability during experimental conditions. We investigate the diffusion characteristics of His-GFP in the shell compared to the diffusion of the same complex in GUVs and the specificity of the loading of His-GFP on the MB shell. We also demonstrate the scope of potential payloads of the NTA(Ni)-functionalized MBs by decorating them with three different His-tagged proteins and a His-tagged small molecule dye. Subsequently, we assess the use of these MBs to tag the plasma membrane of adherent A549 cells with His-GFP with and without US exposure using confocal fluorescence microscopy. Lastly, using confocal microscopy and flow cytometry, we investigate this “membrane tagging” effect with MBs decorated with the targeting protein His-transferrin, to specifically target A549 cells expressing the transferrin receptor. This study lays the groundwork for the potential use of custom-made protein-decorated MBs in immunomodulation, which could provide an inexpensive, low-risk alternative to *ex vivo* therapies.

## 2 Results and Discussion

### 2.1 Protein-Decorated MB Characterization

#### 2.1.1 Population Statistics and Experimental Stability

**Figure 1:**
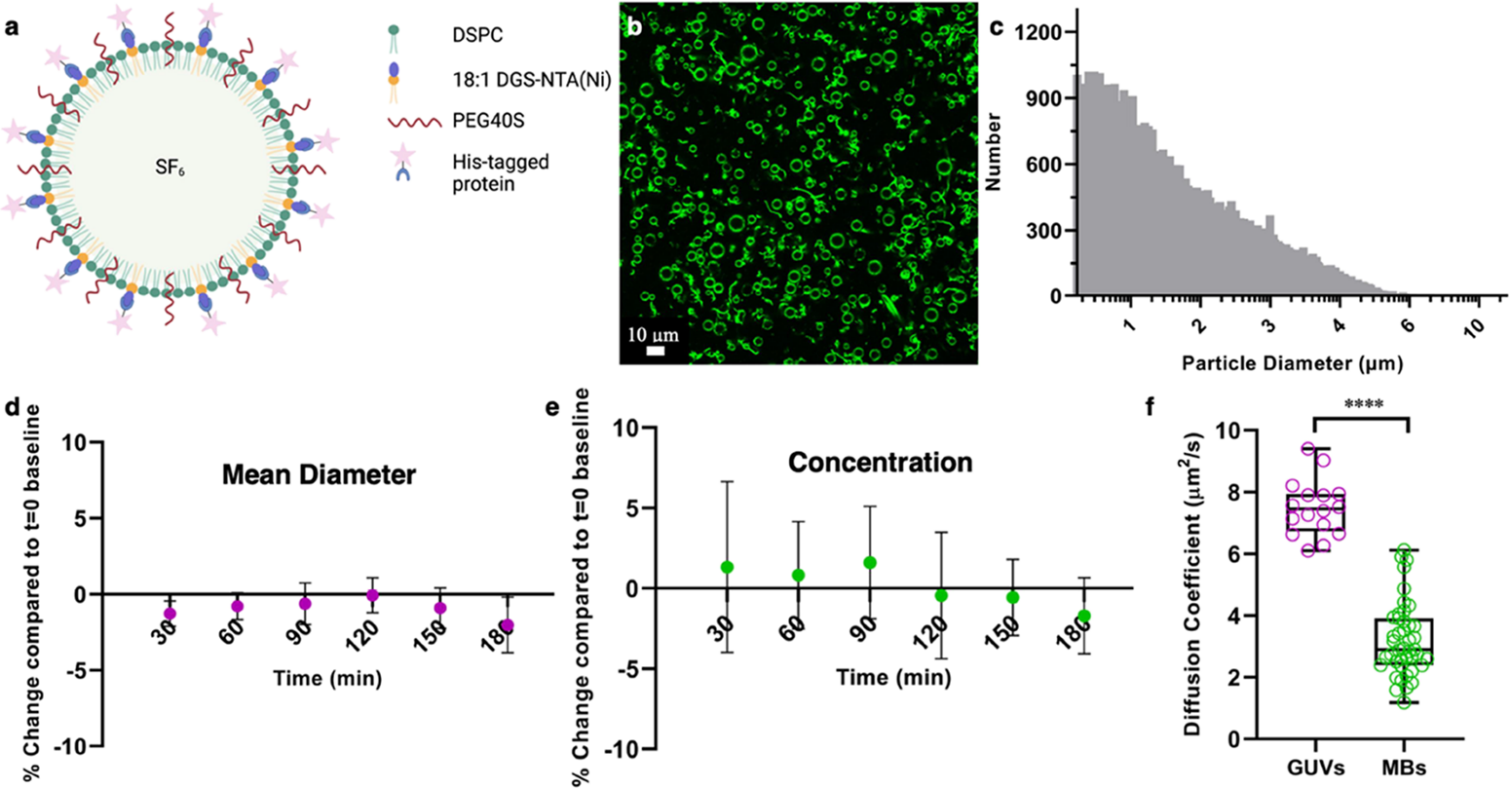
His-GFP-functionalized DSPC-PEG40S-DGS-NTA(Ni) MBs, fabrication and characterization. (a) Schematic of the proposed MB consisting of DSPC (blue), PEG40S (yellow), DGS-NTA(Ni) (orange), with an SF_6_ core and His-GFP decoration (created with BioRender.com). (b) Representative confocal fluorescence microscopy image of His-GFP-functionalized MBs (green) obtained at 40× magnification. The data shown are representative of one MB sample. (c) Representative MB size distribution histogram (number of events = 48,244), showing a mean diameter of 1.99 ± 1.21 μm and a concentration of 4.82 × 10^9^ bubbles per mL, obtained with the multisizer. Stability results in terms of the mean diameter (d) and concentration (e) for DGS-NTA(Ni) MBs functionalized with His-AF488 (*N* = 3). Stability statistics were obtained at 30 min intervals for a duration of 180 min. Shown is the mean percentage deviation from the baseline (*t* = 0 min) mean diameter (d) or concentration (e) histograms, respectively. The error bars represent the standard deviation. (f) Using FCS, the diffusion of the His-GFP Ni-lipid conjugate in MBs was investigated and compared to the diffusion of the same conjugate in giant unilamellar vesicles (GUVs). The conjugate is mobile in both GUVs and MBs, though slower in MBs (Mann–Whitney test, *p* < 0.0001).

The first objective was to design, fabricate, and characterize the protein-decorated MBs. They consist of 1,2-distearoyl-*sn*-glycero-3-phosphocholine (DSPC), polyoxyethylene (40) stearate (PEG40S), and 1,2-dioleoyl-*sn*-glycero-3-[(N-(5-amino-1- carboxypentyl)iminodiacetic acid)succinyl] (nickel salt) (18:1 DGS-NTA(Ni)) at a molar ratio of 9:0.5:0.2, respectively. Protein-loading takes place through the complexation of NTA(Ni) with His_6_ tags on the proteins (Figure 1a,b). His-GFP and His-tagged Alexa Fluor 488 (His-AF488) were used as a model protein and as a model small molecule, respectively, as they can be easily observed using confocal microscopy.

His-GFP-functionalized MBs were successfully produced at a concentration of ∼10^9^ MBs per mL and a mean diameter of ∼2 μm (Figure 1c). Using confocal microscopy, the fluorescence of His-GFP was investigated to ensure the binding of the protein to the MB shell does not disturb the native conformation of the fluorescent protein, as indicated by maintaining their fluorescent properties (Figure 1b).^27^ To assess the amount of bound fluorescent payload, the loading capacity was assessed using fluorescent Alexa Fluor 488 (His-AF488), the results of which can be found in the Supporting Information. It was found that of the 4 μg mL^–1^ His-AF488 incubated with the MBs, 2.1 ± 0.2 μg mL^–1^ remained on the MBs immediately after fabrication (*N* = 3). Functionality of the cargo protein GFP is maintained during fabrication, indicated by the retention of fluorescence, as visualized by confocal microscopy.

MB stability can be assessed in different ways: (i) the stability of the MBs themselves, in their production conditions, which is important for experimental consistency, and (ii) the effect of biological conditions, such as proteins and ions present in biological fluids, on the stability of the NTA(Ni)–His complex. Regarding (i), both the mean diameter (Figure 1d) and concentration (Figure 1e) of His-AF488-functionalized MBs remain within a 2% deviation from the baseline when stored at 4 °C for up to 3 h (*N* = 3).

#### 2.1.2 Diffusion Characteristics

Using fluorescence correlation spectroscopy (FCS), diffusion of the His-GFP conjugated lipids in the protein-decorated MBs was found to be slower compared to that of the same conjugated protein–lipid complex embedded in GUVs (Figure 1f). However, the His-GFP conjugate in MBs still diffuses with a diffusion coefficient of 3.2 ± 1.2 μm^2^/s, meaning that it is mobile on the MB surface. This mobility is required for material transfer to a target cell plasma membrane.^28^ Moreover, bilayer fluidity can play a role in the formation of high-avidity multivalent bonds between histidine residues and Ni head groups.^29^

#### 2.1.3 Specificity and Variety of Payloads of NTA(Ni)-MBs

**Figure 2:**
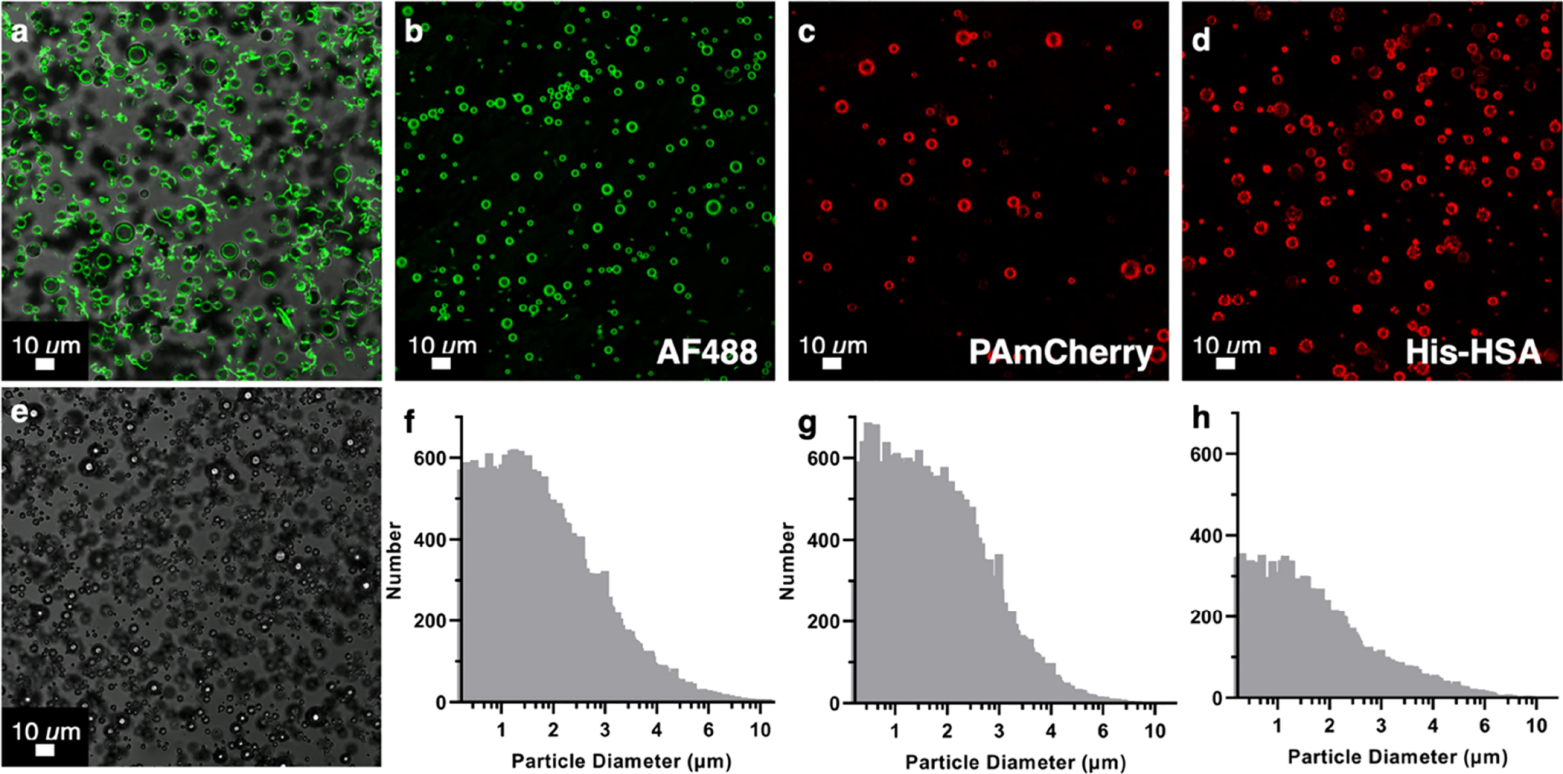
Confocal fluorescence microscopy images of His-GFP incubated (a) functionalized DSPC-PEG40S-DGS-NTA(Ni) MBs and (e) control DSPC-PEG40S MBs. As can be observed, there is no nonspecific binding of His-GFP (green) to MBs lacking nickelated lipid (e). In contrast, His-GFP binds specifically to nickelated MBs (a). To demonstrate the wide applicability, MBs were also functionalized with His-AF488 (b), His-PAmCherry (c), and His-HSA (d). Both confocal microscopy images and MB size distributions were obtained. The statistics are His-AF488:4.32 × 10^9^ MBs mL^–1^, 1.92 μm mean diameter (f); His-PAmCherry: 3.48 × 10^9^ MBs mL^–1^, 2.20 μm mean diameter (g); His-human serum albumin: 2.08 × 10^9^ MBs mL^–1^, 1.89 μm mean diameter (h).

The specificity of His-GFP for MBs containing DGS-NTA(Ni) was investigated using confocal fluorescence microscopy, where DGS-NTA(Ni) containing bubbles were compared to DSPC-PEG40S bubbles, which were made according to the same protocol as the DGS-NTA(Ni)-functionalized MBs, with a molar ratio of 9:0.5 for DSPC and PEG40S, respectively. The specificity of this binding is demonstrated, as His-GFP (green) binds to MBs containing the DGS-NTA(Ni) lipid (Figure 2a), whereas no binding can be observed for the control bubbles without the nickelated lipid (Figure 2e).

To explore the potential of varying the payload on these DGS-NTA(Ni)-functionalized MBs, aside from His-GFP and His-AF488 (Figure 2b,f), His-PAmCherry (Figure 2c,g) and His-tagged human serum albumin (His-HSA) (Figure 2d,h) MBs were also fabricated.

### 2.2 Proof-of-Principle Protein–Lipid Complex Transfer to the Cell Membrane In Vitro

**Figure 3:**
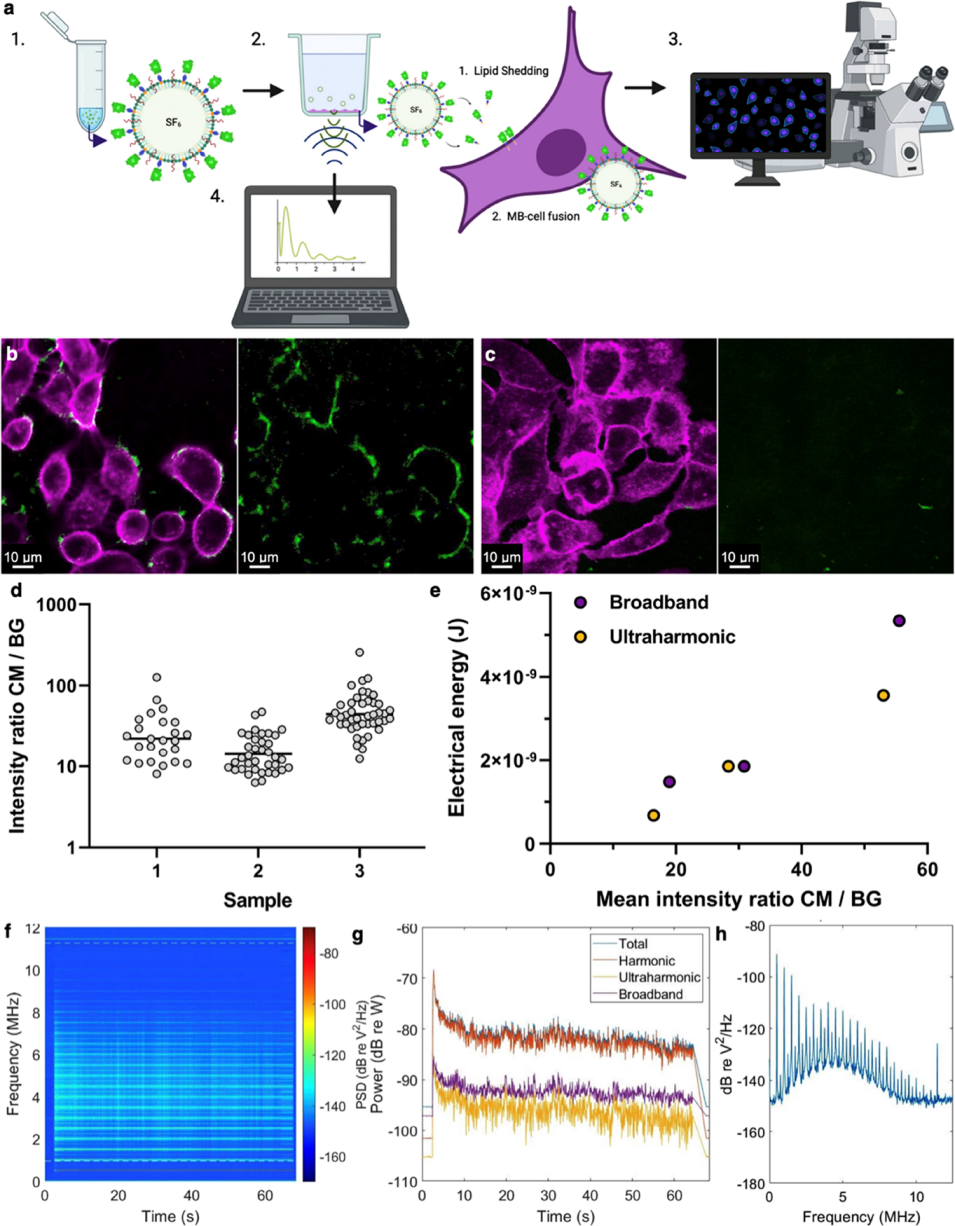
(a) Schematic overview of the conducted experiment. His-GFP microbubbles are formulated (1), A549 cells are incubated with 1 × 10^8^ MBs mL^–1^ and exposed to US (2), the “membrane tagging” either through lipid shedding or MB–cell fusion is assessed using confocal microscopy (3), and the acoustic behavior of the MBs is registered using a PCD and analyzed using MATLAB (4) (created with BioRender.com). (b) Example confocal microscopy images of A549 cells stained with Cell Mask Deep Red (magenta) and His-GFP NTA(Ni) lipids (green) after 60s of US exposure (0.5 MHz, 200 kPa PNP, CW), with the second panel only showing the green channel. (c) Example no US control, with the second panel only showing the green channel. A qualitative difference in the amount and pattern of transfer/fusion of His-GFP NTA(Ni) lipid to the cell membranes can be observed. (d) Intensity of the His-GFP complex within the cell membrane (CM) compared to the background (BG) for three samples (ANOVA, *p* < 0.0001). (e) Total electrical energy for the broadband signal (purple) and ultraharmonic signal (yellow) for each observed mean intensity of the His-GFP complex within the cell membrane (CM) compared to the background (BG). Broadband points are nudged right to avoid overlapping data. (f) Example spectrum histories over a 60 s exposure of a suspension of His-GFP at 1 × 10^8^ MBs mL^–1^, with (g) full spectra and total, harmonic, ultraharmonic, and broadband signal powers, all as a function of time. (h) Frequency domain PCD trace corresponding to the onset of the US signal. Drive conditions were 0.5 MHz, 0.2 MPa peak negative pressure, continuous wave, a 60 s total exposure duration.

After the successful fabrication and characterization of the protein-decorated MBs, the potential of utilizing these bubbles to tag cell membranes by using US was investigated. The experiments were carried out in a system for acoustic transfection (SAT), which consists of a cell exposure compartment, an US source (0.5 MHz), and a single-element transducer functioning as a passive cavitation detector (PCD), all of which are integrated into a benchtop test chamber. MB material transfer to the cell membranes of adherent A549 cells under US exposure (0.5 MHz, 60s CW, 0.2 MPa PNP) was assessed using confocal microscopy, and MB acoustic behavior was registered using passive cavitation detection and analyzed using custom-written MATLAB code (Figure 3a).

His-GFP-functionalized NTA(Ni) lipid transfer to and/or MB fusion with the cell membrane occurs following US exposure (Figure 3b), a process that is minimally observed in the control samples, which were not exposed to US (Figure 3c). These results align with previous work by Carugo et al., which showed that lipid transfer/MB fusion was substantially enhanced by US exposure.^21^ More example field-of-views can be seen in Figure S1. As a negative control, we have verified that free GFP does not bind to the cell surface by itself (Figure S2), and hence that fluorescence signal detected on the cell surface is the result of interaction with MBs functionalized with GFP. Functionality of the cargo protein GFP is maintained not only during fabrication of the MBs but also during experimental handling and posttherapeutic US exposure, indicated by the retention of fluorescence as visualized by confocal microscopy in Figure 3.

To quantify the membrane tagging efficiency, we used the fluorescence intensity of the GFP signal in the membrane divided by the background fluorescence intensity. This intensity ratio would be equal to 1 for no tagging and increase with a higher tagging efficiency. This ratio, despite varying from sample to sample, was above 10 for all replicates (Figure 3d). The mean intensity appears to correlate with the recorded ultraharmonic and broadband cavitation energies recorded (defined as odd integer multiples of half the fundamental frequency (*f*_0_/2) and inertial cavitation, respectively) (Figure 3e). This shows that increasing bubble activity (represented by ultraharmonic and broadband energies) leads to an increase in membrane tagging. The example spectrum histories in Figure 3f–h show the presence of this elevated and prolonged broadband component, which might also have contributed to the diffuse rather than punctate His-GFP-functionalized NTA(Ni) lipid deposition on the cell membranes in Figure 3b.

### 2.3 Targeting of Protein-Decorated MBs to Cell Membranes

To improve the selectivity of protein delivery and limit off-target interactions by selectively directing MBs to tissues of interest, such as the vasculature, immune cells, or cancer cells, MBs can be conjugated with targeting ligands, e.g., peptides and antibodies, on their surface through a variety of strategies, including the incorporation of phosphatidylserine,^30,31^ the noncovalent (strept)avidin binding to biotin,^32,33^ and covalent reactions such as amide coupling, azide–alkyne cycloaddition, and thiol-maleimide addition.^34,35^

We tested whether we could use our systems for receptor targeting. As a lung cancer cell line, A549 cells express high levels of the transferrin receptor (TfR),^36^ providing a convenient target for functionalized MBs, which was experimentally verified as shown in Figure S3. Transferrin (Tf) and TfR play a critical role in cellular iron uptake, which is required for various processes involved in cell proliferation. For this reason, cancer cells often overexpress TfR, and their overexpression is often associated with a poor prognosis. The expression of TfR modulates the proliferation, migration, invasion, and metastasis.^37,38^ For example, increased expression of the TfR is seen in certain types of breast cancer,^39^ ovarian cancer,^40^ and lung cancer,^41^ as well as blood malignancies such as leukemia.^42^ The overexpression of the transferrin receptor on malignant cells, its role in cancer cell pathology, the extracellular accessibility, and the receptor’s role in internalization makes the transferrin receptor a useful system for proof-of-principle targeting studies, as shown in previous work on liposome-bubble delivery systems.^43,44^

**Figure 4:**
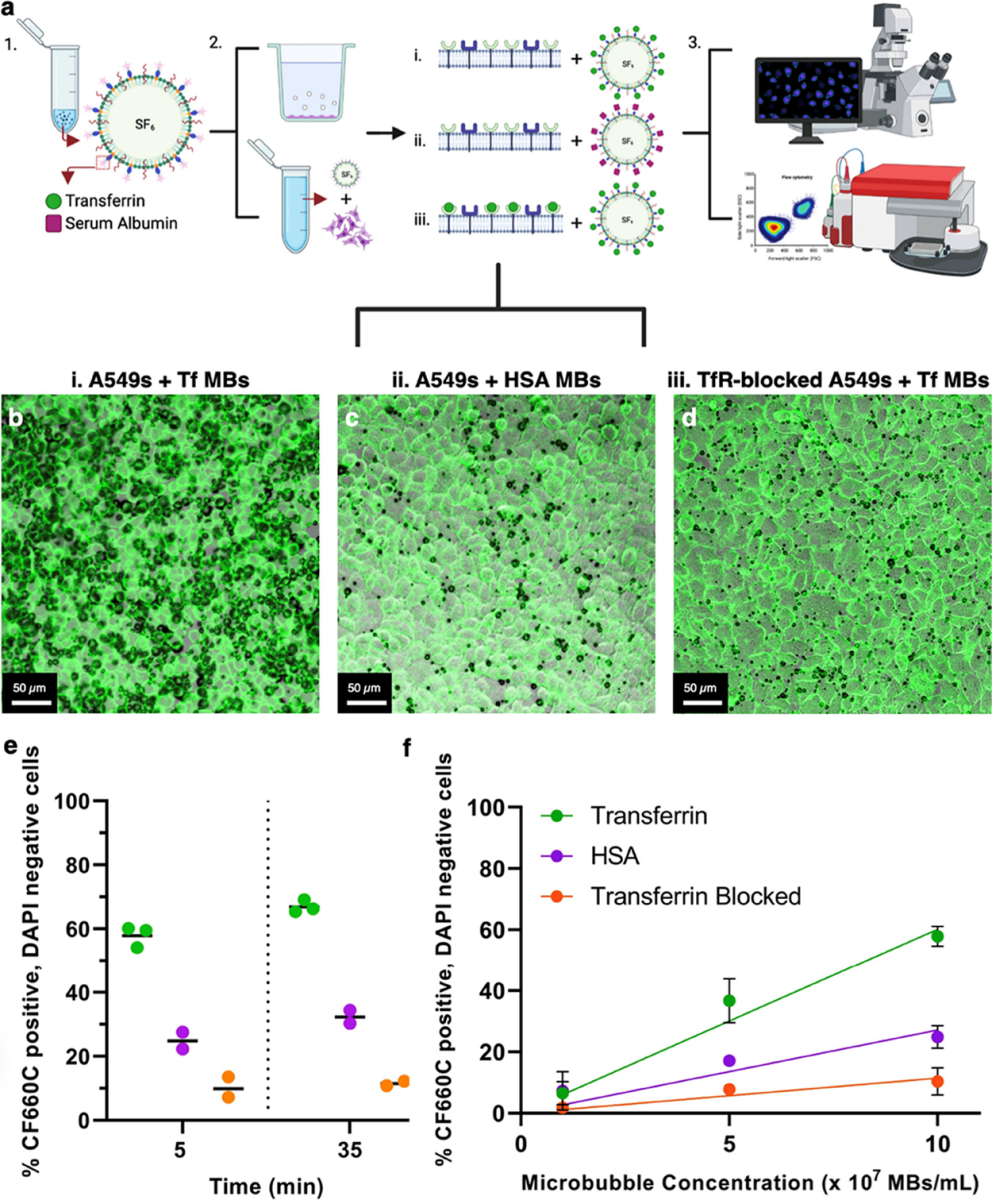
(a) Schematic overview of the experiment. His-transferrin (Tf) and His-human serum albumin (HSA) MBs are formulated (1), and three treatment conditions are explored for both adherent A549 cells and A549 cells in suspension: Tf-MBs with regular A549 cells (2.i), HSA-MBs with regular A549 cells (2.ii), and Tf-MBs with TfR-blocked A549 cells (2.iii), (3) MB–cell attachment and subsequent material transfer are assessed using confocal microscopy for adherent cells and flow cytometry for cells in suspension (created with BioRender.com). Representative 10× confocal microscopy images of A549 cells (green, stained with CellMask Green) and MB PMT overlays (black, brightfield-like) of His-transferrin functionalized MBs (b) and His-human serum albumin MBs (c) with non-TfR-blocked A549 cells. (d) To quantify the amount of nonspecific binding, MB–cell attachment for His-transferrin-functionalized MBs with TfR-blocked A549 cells was assessed. (e) MB–cell attachment and MB material transfer of His-transferrin and His-human serum albumin for non-TfR- and TfR-blocked A549 cells at 1 × 10^8^ MBs mL^–1^ (50 MBs per cell). The % of A549 cells that are positive for CF660C, the fluorophore with which both transferrin and human serum albumin are labeled, and negative for DAPI are shown, indicating a difference between both His-transferrin (green, *N* = 3) and His-human serum albumin (magenta, *N* = 2)-functionalized MBs with non-TfR-blocked A549 cells, and His-transferrin MBs with blocked transferrin receptor A549 cells (orange, *N* = 2). (f) Material transfer efficiency as a function of MB concentration for all three MB–cell experimental conditions at three MB concentrations: 1 × 10^8^ MBs mL^–1^ (50 MBs per cell), 5 × 10^7^ MBs mL^–1^ (25 MBs per cell) and 1 × 10^7^ MBs mL^–1^ (5 MBs per cell). The lines represent linear regression fits [transferrin slope 6.014 × 10^–7^ %/(MB mL^–1^), *p* < 0.0001; HSA slope: 2.713 × 10^–7^ %/(MB mL^–1^), *p* = 0.004; transferrin-blocked slope 1.144 × 10^–7^ %/(MB mL^–1^), *p* = 0.0008].

The targeting of His-Tf-decorated MBs to A549 cells was investigated qualitatively using confocal microscopy and quantitatively using flow cytometry and compared to two control conditions: (i) His-HSA MBs with A549 cells and (ii) His-Tf MBs with TfR-blocked A549 cells (Figure 4a). The former control condition shows receptor-specific binding for the human serum albumin receptor, which is present, albeit not upregulated, in A549 cells. The latter control condition was chosen to assess the amount of nonspecific binding or tethering of the MBs to the cells^45^ by intervening in the protein-receptor pathway through blocking of the binding sites.

Qualitatively, His-Tf MBs were found on A549 cells (Figure 4b) in larger quantities than both His-HSA MBs on A549 cells (Figure 4c) and His-Tf MBs on TfR-blocked A549 cells (Figure 4d). No distinct difference can be observed between the last two conditions from these confocal images. To ensure that the His-Tf MBs were specifically attached to the cells/cell membranes, a higher magnification was used (40× objective). Figure S4 shows an example of 40× magnification for each treatment condition, with MBs and MB deposits present on cell membranes; the His-Tf MBs and MB lipid fragments, leftover after the dissolution of the MBs, were deposited on the cell membrane. This predominant binding of Tf MBs to cells which do not have the TfR blocked provides evidence for the functionality of the Tf protein being unaffected through either the fabrication process or subsequent experimental handling.

To assess the effect of His-Tf targeting of the MBs quantitatively, MBs and cells were mixed in transwells and subsequently analyzed using flow cytometry, the raw data of which can be found in Figures S5 and S6. A comparison was made with His-HSA-decorated MBs, at three different MB concentrations: 1 × 10^8^ MBs mL^–1^ (50 MBs per cell), 5 × 10^7^ MBs mL^–1^ (25 MBs per cell), and 1 × 10^7^ MBs mL^–1^ (5 MBs per cell). The cells of interest were defined as cells in population 1—identified as the live cell population, which were negative for 4′,6-diamidino-2-phenylindole (DAPI)—here used as a marker of permeabilization, similar to propidium iodide—and positive for CF660C, with which the targeting proteins are labelled. Measurements were performed at *t* = 5 and *t* = 35 min after incubation of cells with MBs to assess the possible effect of time on both cell viability (Figure S7) and MB material transfer (Figure 4).

For 50 MBs per cell, as shown in Figure 4e, the fraction of cells that are positive for CF660C-labeled His-Tf is 67%, compared to 32% of cells being positive for CF660C-tagged His-HSA and 12% of blocked cells being positive for CF660C-labeled His-Tf, at *t* = 35 min, resulting in observable differences between these three conditions at both time points. Time also appears to have an effect, as these numbers increased from 58, 25 and 10%, respectively, from *t* = 5 min. A possible reason for the higher percentage of cells positive for MB material transfer from His-HSA MBs compared to the control condition of His-Tf MBs with blocked cells might be that HSA might bind nonspecifically to cell surface or A549 cells do express human serum albumin receptors.^46,47^

To study the relationship between the number of MBs per cell and the fraction of cells that are “membrane tagged”, two more MB concentrations were investigated: 5 × 10^7^ MBs mL^–1^ (25 MBs per cell) and 1 × 10^7^ MBs mL^–1^ (5 MBs per cell). The results show an approximately linear relationship for each MB–cell experimental condition, as shown in Figure 4f, which can be used to determine the concentration of MBs needed to achieve a certain percentage of material transfer or “membrane tagging”.

## 3 Implications and Limitations

This work demonstrates the successful fabrication and characterization of protein-decorated MBs by using the interaction between NTA(Ni) and His-tags. Advantages over other MB ligand-loading methods of the NTA(Ni)–His complex, such as streptavidin/avidin–biotin interaction and maleimide–thiol addition,^48^ include site-specific binding and limited to no immunogenicity. Notable downsides to this strategy include the potentially limited in vivo stability upon intravenous administration due to the competition of histidine-rich serum proteins with His-tagged proteins with which the MBs are decorated,^49^ and therefore extending the stability study to both whole blood and in vivo conditions is important to assess in future work.

Another possible hurdle toward the translation to an in vivo application is the potential cytotoxicity from the presence of nickel. To address this, ICP-OES single-element spectroscopic analysis was performed by Medac Ltd. on both the lipid film prior to MB production and on freeze-dried samples of NTA(Ni)-functionalized MB showing at least a 16-fold decrease in nickel concentration in the bubble samples compared to the precursor lipid film (section S9 in the Supporting Information). This amounts to concentrations of Ni in one bubble batch (total volume 1 mL) of 0.70 μg mL^–1^, and subsequent potential serum concentrations of less than 0.2 ng mL^–1^, which is 10-fold lower than potentially dangerous nickel serum concentrations of 2.0 ng mL^–1^ or even toxic concentrations of 10 ng mL^–1^ as indicated by the Mayo Clinic. Furthermore, this model system is in its current form not able to transfer whole membrane proteins; i.e., it is not able to transfer transmembrane and intracellular domains.

Potential avenues to be explored to further optimize the His-tagged-based model system of these protein-decorated MBs for in vivo applications include (i) increasing the binding affinity by replacing NTA with tris-NTA^26,50^ or anti-His_6_ antibodies, (ii) increasing the surface density of NTA(Ni) on the MBs, (iii) using a polyethylene glycol linker between the phospholipid headgroup and metal complex, and (iv) changing the length and saturation of the phospholipid acyl chains. General optimization steps to be taken regarding MB production include the selection of filling gas or coating materials,^51,52^ or formulating the MBs as volatile nanodroplets^53^ to improve their in vivo stability and circulation time.

These experiments were performed with cells adhered to a polymer membrane boundary. It is known that the presence of a boundary affects the response of MBs.^54,55^ MB behavior near a surface includes asymmetric oscillations, microstreaming, and microjetting at sufficiently high pressures. Moreover, the phase difference between the reradiated sound field and the sound field reflected of the boundary, and thus the net direction of the MB movement, is determined by the elasticity of said boundary.^13^ For this reason, observations made in conventional in vitro set-ups, like the one presented here, may not represent the additional complexities of in vivo situations, such as the heterogeneous nature of tissues.

Similar note should be taken regarding targeting selectivity. In future experiments, targeting efficiency and lipid transfer should be assessed in a coculture experiment, in the presence of serum, with a cell line expressing the receptor of interest, e.g., the transferrin receptor, and at least one cell line that does not. Furthermore, it should be noted that not only cancer cells exhibit high levels of the transferrin receptor on their cell membrane but also healthy cells, such as liver cells,^56^ express this receptor. Thus, this model protein might not be suitable for clinical translation due to the risk of off-target toxicity, and alternative targeting proteins may be required.

Additionally, fluorescent model proteins were chosen to be suitable for this proof-of-principle study of membrane tagging by protein-functionalized MBs and US, including His-GFP and fluorescent His-transferrin, which were shown to be functional based on their fluorescence and ability to bind A549s (Figure S3), respectively. Proposed future work, therefore, includes biologically relevant proteins and antibodies to improve targeting efficiency and confirm biological functionality and activity, e.g., entities that target immune cell receptors, including CD3,^57^ CD4,^58^ CD7,^59^ and CD8,^60^ or diseased cells, including CAR-T cell therapy targets CD19 for B-cell and follicular lymphoma^61^ or B-cell maturation antigen for multiple myeloma.^62^

## 4 Conclusions

In this study, protein-decorated MBs were created and used to “tag” cell membranes of interest with a model protein and to investigate US-mediated MB–cell membrane interactions. Confocal microscopy confirmed protein–lipid material transfer to adherent A549 cell membranes and that this was significantly promoted by exposure to US. Quantitatively, this “membrane tagging” effect was positively correlated with the MB activity as measured in terms of ultraharmonic and broadband cavitation signal energy. Lastly, these protein-decorated MBs were modified to selectively bind MBs to cells of interest to improve the selectivity of the proposed “membrane tagging” and limit potential off-target interaction. MBs were functionalized with His-Tf, as A549 cells overexpress transferrin receptors on their outer membranes. The results showed a significant increase in MB–cell attachment and subsequent “membrane tagging” of His-Tf MBs compared to control His-HSA MBs. There was also limited binding of His-transferrin-functionalized MBs to A549 cells with transferrin receptors blocked by excess free transferrin, confirming binding specificity. This work demonstrates the potential use of custom-made protein-decorated MBs to “tag” target cell membranes with proteins and, if successfully developed, may have a role in immunomodulation and provide an inexpensive, low-risk alternative to CAR-T cell therapy.

## 5 Experimental Details

### 5.1 Protein-Decorated MB Fabrication

A batch sonication protocol was employed to prepare the MBs.^21^ In brief, the protocol is as follows: 1,2-distearoyl-*sn*-glycero-3-phosphocholine (18:0 PC (DSPC), Avanti Polar Lipids, UK), polyoxyethylene (40) stearate (PEG40S, Sigma-Aldrich, UK), and 1,2-dioleoyl-*sn*-glycero-3-[(N-(5-amino-1-carboxypentyl)iminodiaceticacid)succinyl] (nickel salt) (18:1 DGS-NTA(Ni), Avanti Polar Lipids, UK) were dissolved in chloroform (Sigma-Aldrich, UK) and mixed in a glass vial at a molar ratio of 9:0.5:0.2, containing 20 mg of lipid constituents in total. The chloroform solution was covered with perforated parafilm (Bemis Company, Inc., Neenah, WI, USA) and subsequently heated overnight on a hot plate set to 50 °C, facilitating evaporation of chloroform and the formation of a homogeneous lipid film.

The obtained dry lipid film was suspended in 2 mL of Dulbecco’s phosphate-buffered saline (DPBS, pH 7.4, Life Technologies, Paisley, UK) and stirred at 100 °C on a magnetic stirrer hot plate for a minimum of 45 min. Lipids were then homogeneously dispersed for 150 s using a sonicator (Microson XL 2000, probe diameter 3 mm, 20 W, 22.5 kHz, Misonix Incorporated, NY, USA) with the tip completely immersed in the lipid solution (power setting 3). MBs were subsequently formed by placing the sonicator tip at the air–water interface under constant sulfur hexafluoride flow (The BOC Group plc, UK) and sonicated for 30 s to create a cloudy suspension of MBs (power setting 14). Immediately after production, the vial with the MB suspension was capped and placed on ice for approximately 5 min prior to the first washing step. MBs were kept on ice during all subsequent steps, except during ligand incubation.

MBs were washed once to eliminate the excess free NTA(Ni)-functionalized lipids using a centrifugation method without size isolation. In summary, MBs were loaded into a 2 mL syringe and centrifuged for 5 min at 300*g* (Denley, BR401, UK). Following centrifugation, the subnatant was discarded, and MBs were resuspended in 1 mL of DPBS solution containing His_6_-GFP (20 μg mL^–1^, Stratech, UK, 27 kDa) or a synthetic equivalent, more cost-effective His_6_-AF488 (4 μg mL^–1^, Cambridge Research Biochemicals Ltd., UK, 1356.4 Da). After 8 min of incubation on a roller mixer (SRT6, Stuart, UK) at room temperature, centrifugation was performed, again for 5 min at 300*g*, to eliminate the excess unbound His-tagged protein and improve the signal-to-noise ratio for fluorescence microscopy experiments, after which MBs were resuspended in 1 mL of fresh DPBS.

### 5.2 MB Characterization: Population Statistics and Experimental Stability

To quantify the MB size and concentration, 10 μL of the produced MB suspension was transferred onto a Neubauer improved cell counting chamber (Hausser Scientific Company) under a 24 × 24 mm glass coverslip (VWR International). Roughly 40 images of MBs were acquired at 40× magnification using a Leica DM500 microscope (Leica Microsystems GmbH, Germany) coupled with a CCD camera (Leica Microsystems GmbH, Germany). MB sizing and counting were performed using purpose-written code in MATLAB.^63^

Population statistics—defined here as MB concentration and mean diameter—were additionally collected using an electrozone sensing approach (Coulter Multisizer 4e, Beckman Coulter, Opa Locka, FI).^64^ In brief, freshly prepared MBs were homogeneously dispersed by gentle agitation. MBs (2 μL) were diluted into 10 mL of Isoton II in an Accuvette. MB samples were characterized three times with thousands of events per repeat. A 20 μm aperture (size range of 0.4–16 μm) was used. For the stability measurements, the MB samples were analyzed over 3 h in 30 min intervals (*N* = 3 at each time point). Approximately 2000–45,000 MBs were imaged per sample depending on initial concentration (6000–135,000 per formulation). For stability analysis, population statistics were obtained as described above over 180 min. From these results, changes in the concentration and size were examined. This was repeated two to three times using a fresh bubble suspension created from a new lipid film each time.

### 5.3 MB Characterization: Specificity

The specificity of His-GFP for NTA(Ni)-containing MBs was investigated using confocal fluorescence microscopy (LSM 780, Carl Zeiss AG, Germany), with magnifications varying from 20× to 63×. Protein-decorated bubbles were compared to control DSPC-PEG40S bubbles (molar ratio: 9:0.5) made according to the protocol described above and thus also incubated with His-GFP. For this purpose, 2 μL of the MB sample (NTA(Ni)-containing MBs or control MBs) was placed on a 75 × 25 × 0.17 mm glass coverslip (Logitech Ltd., Scotland). Excitation of His-GFP was achieved with a 488 nm laser line, and photomultiplier tube (PMT) reflections were simultaneously obtained to resemble brightfield images.

To demonstrate the applicability of DGS-NTA(Ni) MBs to bind a variety of His-tagged dyes and proteins and, therefore, its translational potential, DGS-NTA(Ni) MBs were incubated with 4 μg mL^–1^ of His_6_-AF488 (Cambridge Research Biochemicals Ltd., UK, 1356.4 Da), 20 μg mL^–1^ of His_6_-GFP (Stratech, UK, 27 kDa), 20 μg mL^–1^ of His_6_-PAmCherry (Abcam, UK, 29 kDa), 100 μg mL^–1^ of His_6_-transferrin (SinoBiological, UK, 76.6 kDa), or 100 μg mL^–1^ His_6_-human serum albumin (Abcam, UK, 67 kDa), according to the protocol described above. His_6_-transferrin and His_6_-human serum albumin were fluorescently labeled to allow observation through confocal microscopy and flow cytometry. To this end, the CF660C Protein Labeling Kit (Biotum, UK) was used to label the proteins and, subsequently, the degree of labeling was assessed using a fluorescent plate reader (FLUOstar Omega, BMG Labtech, UK), as per the manufacturer’s guidelines. For His_6_-transferrin and His_6_-human serum albumin, protein recovery was 70 and 100%, respectively, and the degree of labeling was ∼2 dye molecules/protein. The MB samples were subsequently analyzed using confocal microscopy, and their population statics were obtained, as described above.

### 5.4 MB Characterization: Diffusion Characteristics

FCS was employed to study the diffusion characteristics of the NTA(Ni)-His-GFP complex in comparison to the same characteristics in GUVs. GUVs were produced according to a protocol as described by Jenkins et al.,^20^ with 5:5 of POPC and cholesterol. To this end, confocal microscopy (LSM 780, Carl Zeiss AG, Germany) was performed to obtain the experimental FCS curves for both GUVs and MBs by using a 40× water 1.2 NA objective.

After collecting the fluorescence intensity for 10 s with *N* = 3 per MB, using sensitive photodetectors, a hardware correlator correlated the signal from subsequent time points according to the correlation function described in eq 1 to obtain these experimental FCS curves:
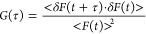
1where the deviation of the measured fluorescence *F*(*t*) from the temporal average value <*F*> is defined as
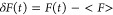
2

These curves were subsequently fitted with eq 3 using FoCuS-point software^65^:
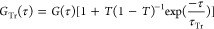
3where *T* is the fraction of molecules occupying the triplet state, τ_Tr_ is the rate of relaxation in the triplet state, and *G*(τ) equals the diffusion fitting function for simple 2D diffusion^66^:
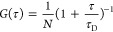
4

to eventually obtain diffusion coefficients for the NTA(Ni)-His lipid–protein complex in the GUVs and MBs. Once the diffusion time *τ*_*D*_ is obtained by fitting the experimental data to eq 5 using FoCuS-point software, developed by Waithe et al. (2016) for super-resolution STED-FCS and time-gated single-photon counting,^65^ the diffusion coefficient *D* can be calculated as follows:
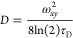
5where ω_*xy*_ is the radial distance of the optical axis, or, in other words, the full width at half-maximum of the focal volume.

To calculate *D*, however, ω_*xy*_ needs to be resolved, which was done by performing a control experiment for the diffusion of freely diffusing Alexa Fluor 488 dye with known diffusion coefficient *D* = 430 μm^2^/s at room temperature and measurable τ_D_, according to the 3D diffusion model^66^:
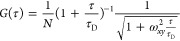
6

### 5.5 Cell Culture

Immortalized human alveolar adenocarcinoma cells (A549 cells) were cultured in T-75 flasks and passaged at 80% confluence (ATCC, UK). A549 growth medium consisted of Dulbecco’s Medium Eagle’s medium supplemented with 10% fetal bovine serum. Cells were grown in a temperature- and CO_2_-controlled incubator at 37 °C and 5% CO_2_. Cells were removed from T-75 culture flasks by exposure to 0.25% trypsin/EDTA for approximately 5 min. Cells were then suspended in 10 mL of culture medium to deactivate the trypsin and subsequently centrifuged for 5 min at 300*g* to form a pellet. Cells were then resuspended in 10 mL of fresh culture medium. Cell concentration and viability were measured using trypan blue and a Countess Automated Cell Counter (Invitrogen, UK). Unless stated otherwise, all cell culture materials were purchased from ThermoFisher Scientific (UK).

For the proof-of-principle experiments, 0.01 × 10^6^ cells were seeded approximately 48 h prior to imaging in 6.5 mm diameter transwells in 24-well plates (Corning, Sigma-Aldrich, UK) in 100 μL of growth medium. For the confocal microscopy targeting experiments, 0.2 × 10^6^ cells were seeded in 35 mm diameter tissue culture-treated Ibidi μDishes (Ibidi, Germany) in 2 mL of the growth medium, 48 h prior to exposure, to obtain near 80% confluence. Prior to treatment, cells were washed twice with DPBS and incubated with 1 μg mL^–1^ of the plasma membrane stain CellMask Deep Red (C10046, Thermofisher, UK) for 8 min. Subsequently, cells were washed twice before incubation with protein-decorated MBs, as specified in the two sections below.

### 5.6 Proof-of-Principle US-Mediated MB–Cell Material Transfer

The US setup was designed to obtain small working volumes of ∼150 μL per sample. A transwell insert is used (6.5 mm diameter, Corning) on which cells are grown. The transwells have a permeable membrane and must be placed in cell media or DPBS rather than water. The system for acoustic transfection (SAT) builds upon the prior system development described in Carugo et al.^67^ and is shown in detail in Gray et al. as SAT3.^68^ The orientation of this setup is such that MBs are situated above the cell layer; thus, interaction between the MBs and cells is not aided by the buoyancy of the bubbles.

The setup’s internal volume is approximately 7.6 L. An internal chamber of 0.3 L was added to minimize the disposable volume and allow biologically relevant fluids other than the tank fill water to be used (e.g., cell culture media or DPBS). The internal chamber bottom is made from a 30 μm-thick mylar sheet to allow maximum acoustic transmission. The SAT3 compartment is filled by syringe or pipet and sealed by press-fitting a rubber stopper/bung (6 mm bottom diameter and 8 mm top diameter, 391-2101, VWR).

The US source used in the SAT3 is a 0.5-MHz focused source with a main lobe width that matches the diameter of the cell attachment area (0.5 MHz, H107 with central hole, Sonic Concepts), and it requires an impedance matching network (H-107, Sonic Concepts) between the amplifier and the transducer. Experiments were performed at 37 °C to mimic physiological conditions, established by using an aquarium heater (EasyHeater, 100 W, Aquael). The acoustic field is terminated in a fixed boundary to eliminate variability from air–water interfaces in partially filled chambers. This was accomplished by installing an acoustic absorber (1 cm thickness, APTFlex F28, Precision Acoustics) on the chamber lid to further reduce the field complexity that may arise from boundary reflections.

To allow for compact configuration, the PCD (5 MHz center frequency, IBHGO54, and Olympus NDT) is integrated into the US source base. The reradiated field is picked up by using a 90° reflector (F-102, Olympus NDT). The response of the PCD to the US source was minimized by selecting a PCD with a center frequency at least five times that of the US source and passing the signal through a 1.8 MHz high pass filter (E508, Thorlabs, UK). The signal was then passed through a preamplifier (SR445A, Stanford Research Systems), used for 5x amplification to ensure capture of the smallest expected signals and improve the signal-to-noise ratio of the result.

A digitizer was employed as a 12-bit streaming USB oscilloscope (HS5-110-XM, TiePie Engineering) to record the signal after it was passed through a 50-ohm impedance matching network. The resultant PCD traces were processed using custom-written MATLAB code to obtain power spectra and an indication of the various components, e.g., broadband, harmonics, and ultraharmonics, making up the signal. Harmonics are defined as integer multiples of the fundamental frequency or center frequency of the transducer (*f*_0_), ultraharmonics are defined as the odd integer multiples of *f*_0_/2, and broadband noise is defined as inertial cavitation and equals the total signal minus the harmonic and ultraharmonic components.

To minimize the likelihood of cavitation in the propagation path, the filtered water, functioning as the fill liquid, was degassed under a pressure of −10^5^ Pa for at least 2 h in situ. Any residual bubbles were cleared from the transducer and media container surfaces immediately after filling and again just before exposure experiments. The chamber was given sufficient time to heat at 37 °C, verified with a thermometer, before commencing experiments. The US source power amplifier was allowed to warm so that the gain and output were stable with respect to time. The transwells with A549 cells grown on the membrane were filled with 200 μL of MB suspension (1 × 10^8^ MBs mL^–1^).

The cell exposure compartment was formed by carefully sealing the transwell with a rubber plug and removing any overflow liquid with a clean paper towel or wipe. The compartment was checked for evidence of entrapped macrobubbles, and if present, the above steps were repeated. The cell exposure compartment was then press-fitted into the compartment holder in the chamber lid and subsequently placed in the SAT3 chamber, lowering the lid at an angle to avoid the formation of macrobubbles on top of the absorber and holder. US parameters were based on previous experiments as performed by Carugo et al.,^21^ e.g., 0.5 MHz center frequency, 200 kPa peak negative pressure (PNP), continuous wave (CW), and a 60 s total exposure duration. The calibration uncertainty on all pressures is ±12%.

PCD data were recorded without driving the US source for 5 s prior to each US exposure to establish background electronic noise levels and ensure the first exposures were not missed. Experiments were monitored in real time in the time and frequency domains using the TiePie Multichannel software.

Simultaneously, the amplifier output signal that drives the US source was monitored throughout the experiment to ensure that the US source generated the desired pressures. For this purpose, a high voltage probe (PP019 10:1, 250 MHz 12 pF, Lecroy, Teledyne) and an oscilloscope (T3DSO1104 Digital Storage Oscilloscope, Teledyne) were used to visualize the amplifier output.

After US exposure, samples were carefully removed from the test chamber and transferred as required for further analyses, e.g., confocal microscopy. To this end, as described above, cells were stained with a plasma membrane stain, 1 μg mL^–1^ CellMask Deep Red (C10046, Thermofisher, UK), with its excitation maximum at 649 nm and its emission maximum at 666 nm. On the Zeiss LSM 780 system, it is excited by the 633 nm laser, and the emitted signal is picked up between 638 and 755 nm. His-GFP was excited by the 488 nm laser, and the emitted signal is picked up between 493 and 598 nm. PMT reflections were simultaneously obtained to resemble brightfield images.

Due to the porous membrane of the transwells, the obtained images contained considerable amounts of noise in both channels and generally showed reduced sharpness. To combat this, the images were postprocessed in ImageJ to reduce this noise by despeckling.

Using ImageJ, the fluorescence intensity of the protein–lipid deposits on the cell membrane (as a measure of protein delivery) was determined by analyzing the line profile (5 pixels wide) across the membrane and taking the ratio of the peak intensity with the average background intensity to eliminate differences in exposure or processing conditions. This was done for a total of 105 cells spread across three samples at arbitrarily chosen locations across the cell membrane. To investigate whether underlying MB behavior was responsible for the differences in mean intensity across the three samples, the electrical energy (in J) summed across the whole exposure duration was calculated for each signal type (total, harmonic, ultraharmonic and broadband) using custom-written code in MATLAB.

### 5.7 Targeting of Protein-Decorated MBs

The targeting protocol for assessing MB targeting to adherent cells in Ibidi μDishes was as follows. For the confocal microscopy experiments, 0.2 × 10^6^ cells were seeded in 35 mm diameter tissue culture-treated Ibidi μDishes (Ibidi, Germany) in 2 mL of growth medium, 48 h prior to exposure, to obtain near 80% confluence. For flow cytometry experiments, A549 cells were made up to a concentration of 2 × 10^6^ per mL in T-25 cells culture flasks to achieve 4 × 10^5^ cells per SAT3 transwell during experiments. The cells were kept on ice and periodically gently shaken to avoid adherence to the culture flask.

Cells were incubated for 8 min with 1 μg mL^–1^ of the plasma membrane stain CellMask Green (ThermoFisher Scientific, UK). Simultaneously, 12 mL of either His-transferrin or His-human serum albumin MBs was prepared at a final concentration of 2 × 10^7^ MBs mL^–1^ in DPBS. Cells were washed twice with warm DPBS. A PDMS lid was press-fitted to the μDish, and the headspace was subsequently filled with the MB suspension. The μDish was then turned over so that the cells were situated at the top of the dish and placed on a shaker (IKA KS 130 basic, UK). The dish was shaken gently for 5 min at 80 rpm to facilitate contact between the MBs and the cell layer. The MB suspension in the μDish was then replaced with warmed DPBS, and the dish was placed on the shaker the right way up and shaken more violently at 480 rpm for 1 min. This was done so that any nonattached MBs would come off the cell layer. The MB suspension in the μDish was then replaced with 1 mL of warmed DPBS, and the μDish was imaged by a confocal microscope.

To assess the specificity of the binding of His-transferrin MBs to transferrin receptors present on the cell membrane of A549 cells, experiments were carried out with A549 cells, which had their transferrin receptor blocked by free transferrin. To this end, 1.0 × 10^6^ cells in μDishes (∼80% confluence) were incubated with 10 mg of human transferrin (Sigma-Aldrich, UK) for 1 h at 37 °C with 5% CO_2_, as per the protocol specified by Muralidharan et al.^69^

For the suspension experiment, the same free transferrin to cell ratio was maintained, with 10 mg of human transferrin being incubated with 1.0 × 10^6^ cells in suspension for 1 h at 37 °C, while gently shaking every 10 min to promote the mixing of cells and free transferrin and avoid the adherence of the cells to the culture flask. For the flow cytometry experiment, the same free transferrin to cell ratio was maintained, with 10 mg of human transferrin being incubated with 1 × 10^6^ cells in suspension for 1 h at 37 °C, while gently shaking every 10 min to promote the mixing of cells and free transferrin and avoid the adherence of the cells to the culture flask.

For the adherent A549 cells in Ibidi μDishes, qualitative analysis of the targeting ability of His-transferrin MBs was performed using confocal fluorescence microscopy (LSM 780 and LSM 710, Carl Zeiss AG, Germany), with magnifications including 10× (EC Plan-NeoFluar 10×/0.3 M27), 20× (Plan-Apochromat 20×/0.8 M27), 40× (EC Plan-NeoFluar 40×/1.3 Oil DIC M27), and 63× (Plan-Apochromat 63×/1.40 Oil DIC M27).

Cells were stained with a plasma membrane stain, 1 μg mL^–1^ CellMask Green, which has its excitation maximum at 522 nm and its emission maximum at 535 nm and can be analyzed using standard FITC settings on the microscope. On the LSM 780 and LSM 710, it was excited by the 488 nm laser, and the emitted signal is picked up between 493 and 628 nm. His-transferrin and His-human serum albumin, labeled with CF660C, MBs were excited by a 633 nm laser. The emitted signal was picked up between 661 and 759 nm. PMT reflections were simultaneously obtained to resemble brightfield images. Qualitatively, using confocal microscopy, the coating of the cells (green fluorescent) with MBs (black shadows on the PMT reflections) can be observed. Furthermore, the protein–lipid transfer from both His-transferrin and His-human serum albumin MBs to the cell membranes was investigated at 40× magnification.

To obtain quantitative data as well as qualitative results using confocal microscopy, flow cytometry (Beckman Coulter CytoFLEX, USA) was employed to assess the number of cells to which the various MB formulations were attached as well as the viability of the cells after treatment. Each particle was analyzed for visible light scatter and multiple fluorescence parameters. The fluorescence parameters studied included CF660C (ex/em: 667/685 nm) and 4′,6-diamidino-2-phenylindole, dihydrochloride (DAPI) (ex/em: 358/461 nm). DAPI (Invitrogen, ThermoFisher Scientific, UK) was used as a live/dead stain and indicator of permeabilization. DAPI is normally cell-impermeant and can thus be used as a live/dead stain and model drug as its uptake indicates membrane permeabilization, whether due to sonoporation or death. In the interest of investigating the feasibility of targeting MBs to the cell membrane to promote MB material transfer rather than internalization, cells positive for DAPI were excluded from the targeting analysis.

All samples (70 μL of cell suspension in 430 μL of DPBS) were measured at time equals 5 min after MB incubation, allowing for 5 min incubation with 5 μL of 10 μg mL^–1^ DAPI stock solution for each time point (Miltenyi Biotec, DAPI Staining Protocol for FACS). Samples were kept on ice immediately after treatment to slow down degradation and enzymatic activity. It is important to note, however, that keeping the samples on ice also leads to inhibition of transferrin receptor recycling via the endocytotic pathways.^70^ For each sample, 10,000 events (MBs or cells) were analyzed, and appropriate controls (live cells, heated and thus dead/dying cells, and MBs) were carried out prior to commencing the experiment.

The APC-A700, corresponding to CF660C and thus MBs, and PB450, corresponding to DAPI, channel gatings were chosen based on the live cell control sample, which is negative for both CF660C and DAPI. To assess targeting efficiency, the percentage of cells positive for CF660C and negative for DAPI was investigated for both His-transferrin and His-human serum albumin MBs on regular A549 cells and His-transferrin MBs on A549 cells, which had their transferrin receptor blocked by free excess transferrin. The figures show the individual repeats (*N* = 2 or 3) and the mean (black line).

## Supporting Information Available

Additional experimental data, including confocal microscopy images, raw flow cytometry data, and viability data; additional confocal microscopy images of A549 cells and microbubbles showing the effect of ultrasound exposure; representative images of a negative control of A549 cells incubated with GFP or His-GFP; representative images of a positive control of A549 cells incubated with Alexa Fluor 680-labeled His-transferrin; example confocal microscopy images of A549 cells and functionalized microbubbles showing the effect of blocking the transferrin receptor; flow cytometry analysis of His-transferrin and His-human serum albumin functionalized microbubbles; examples of flow cytometry analysis of A549 cells incubated with His-transferrin and His-human serum albumin microbubbles; quantification of cell death produced each microbubble type; evaluation of the loading capacity of His-AF488-functionalized DGS-NTA(Ni) microbubbles; and ICP-OES single-element spectroscopic analysis of microbubble samples (PDF)
