# Supplementary material for: Protein-Decorated Microbubbles for Ultrasound-Mediated Cell Surface Manipulation
Source: ACS Appl Bio Mater. 2023 Dec 4;6(12):5746–58. doi: 10.1021/acsabm.3c00861 (PMC10731656; doi:10.1021/acsabm.3c00861)
Supplement: Supplementary file 1 — mt3c00861_si_001.pdf [file mt3c00861_si_001.pdf]

Supporting Information

Protein-decorated Microbubbles for Ultrasound-Mediated Cell Surface Manipulation

Veerle A. Brans[a], Michael D. Gray[a], Erdinc Sezgin[,b], Eleanor P. J. Stride[*,a]*

[a] Department of Engineering Science, Institute of Biomedical Engineering, University of Oxford, Oxford OX3 7DL, United Kingdom

E-mail: eleanor.stride@eng.ox.ac.uk

[b] Science for Life Laboratory, Department of Women's and Children's Health, Karolinska Institutet, 17165, Solna, Sweden.

E-mail: erdinc.sezgin@ki.se

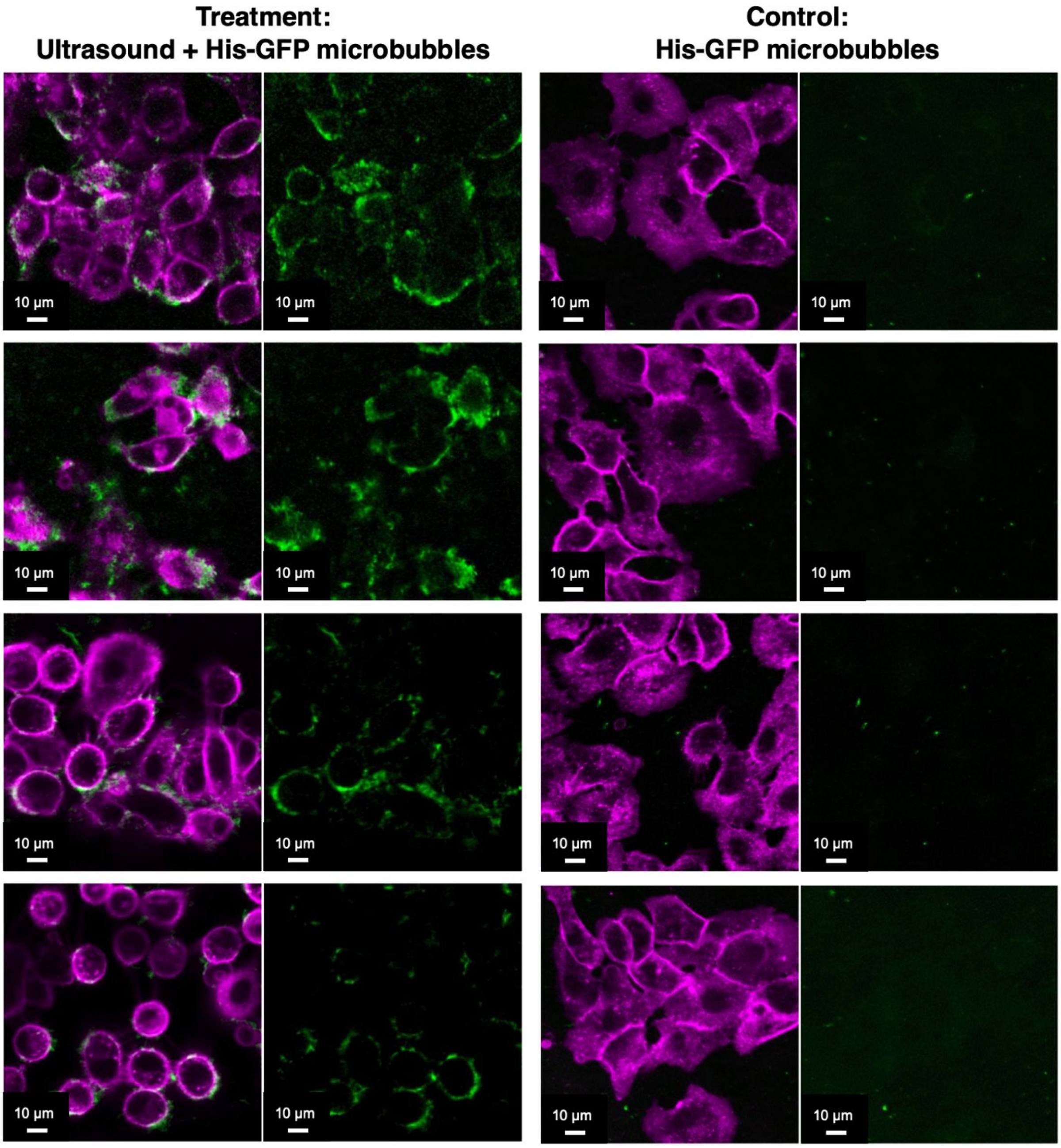

Figure S1. 'Membrane tagging' observed in the SAT3. Additional confocal microscopy images of A549 cells stained with Cell Mask Deep Red (magenta) and His-GFP NTA(Ni) lipids and microbubbles (green) 60s of ultrasound exposure ($N = 3$) (0.5 MHz, 200 kPa PNP, CW) on the left, and corresponding no ultrasound controls ($N = 3$) on the right. A distinct qualitative difference in the amount and pattern of transfer/fusion of His-GFP NTA(Ni) lipid to the cell membranes can be observed.

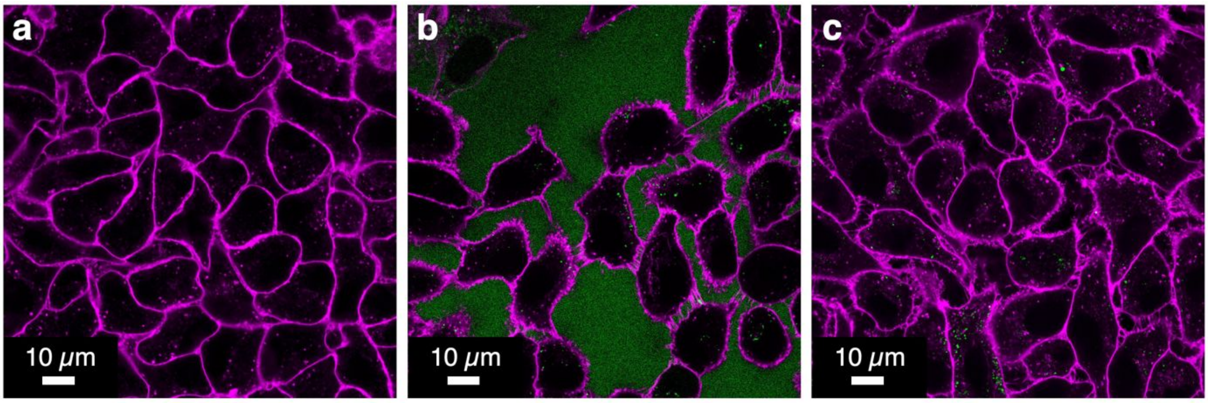

Figure S2. Representative images of a negative control of A549 cells stained with Cell Mask Deep Red (magenta) (a), which are incubated with His-GFP (green) (b) at 20 μg/ml (equal to the maximum possible concentration if all GFP binds to bubbles), clearly showing GFP in the spaces around the cells, not on the cell membrane,as further shown after washing away any excess of His-GFP with DPBS (c).

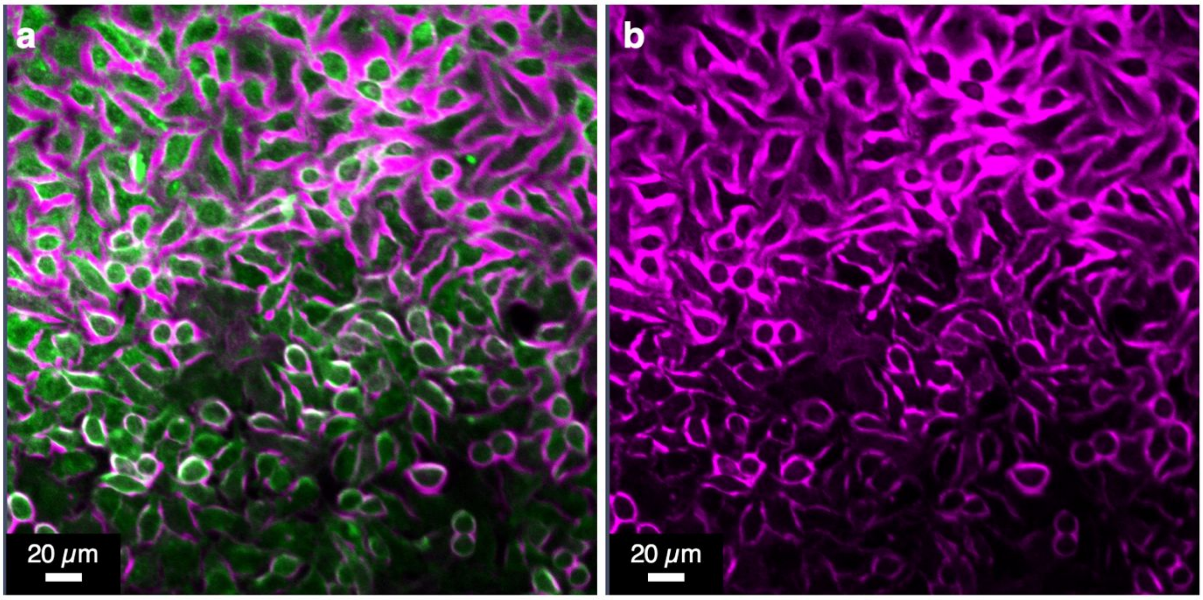

Figure S3. Representative images of a positive control of A549 cells stained with Cell Mask Green (green), which are incubated with Alexa Fluor 680-labelled His-tranferrin (~35 μg/ml) (magenta) (a), clearly showing transferrin bound to the cell membrane, as further visualized by only showing the transferrin channel in (b).

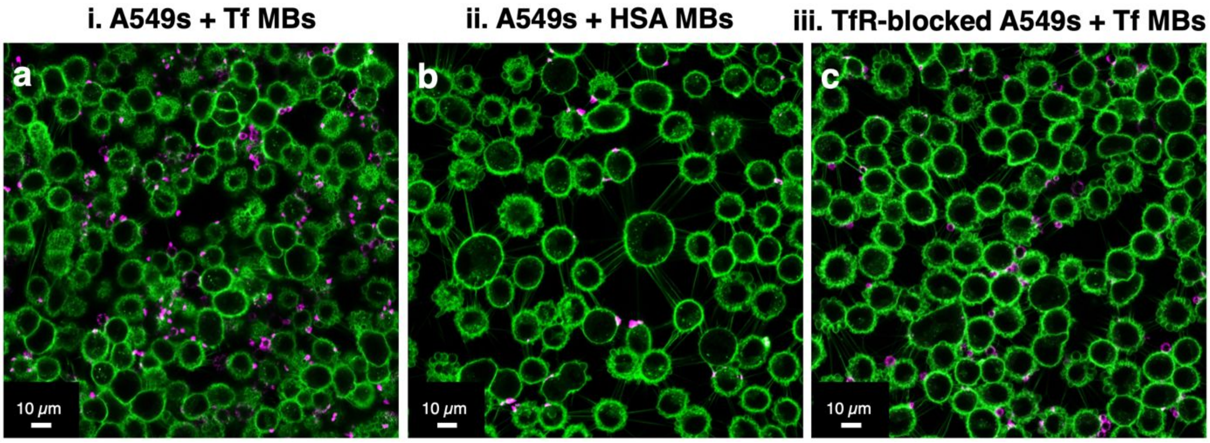

Figure S4. Example confocal microscopy images (40x) of A549 cells (green) and CF^{TM}660C-labelled His-transferrin and His-human serum albumin functionalized microbubbles (magenta) on non-TfR-blocked A549s (a and b, respectively), as well as CF^{TM}660C-labelled His-transferrin microbubbles (magenta) on TfR-blocked A549 cells (c). It shows both microbubbles and microbubble fragment deposits on the cell membrane.

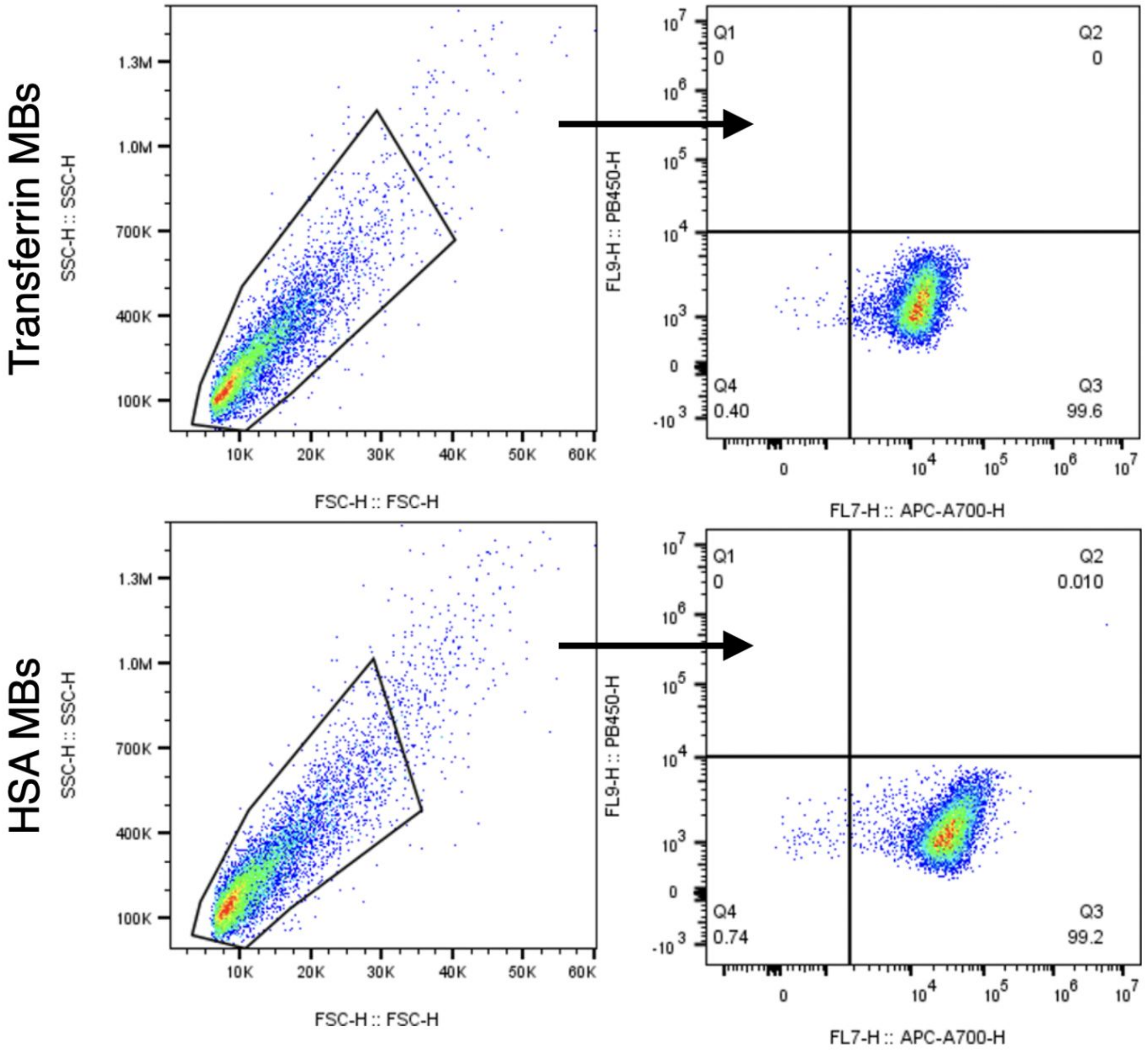

Figure S5. Flow cytometry analysis of His-transferrin and His-human serum albumin functionalized microbubbles. It shows the forward vs side scatter plots and corresponding APC-A700 (CF^{TM}660C) vs PB450 (DAPI) plots. Given the same degree of labeling for both the His-transferrin and His-humans serum albumin, the difference in fluorescence intensity in the APC-A700 channel might be due to a difference in protein loading of the microbubbles.

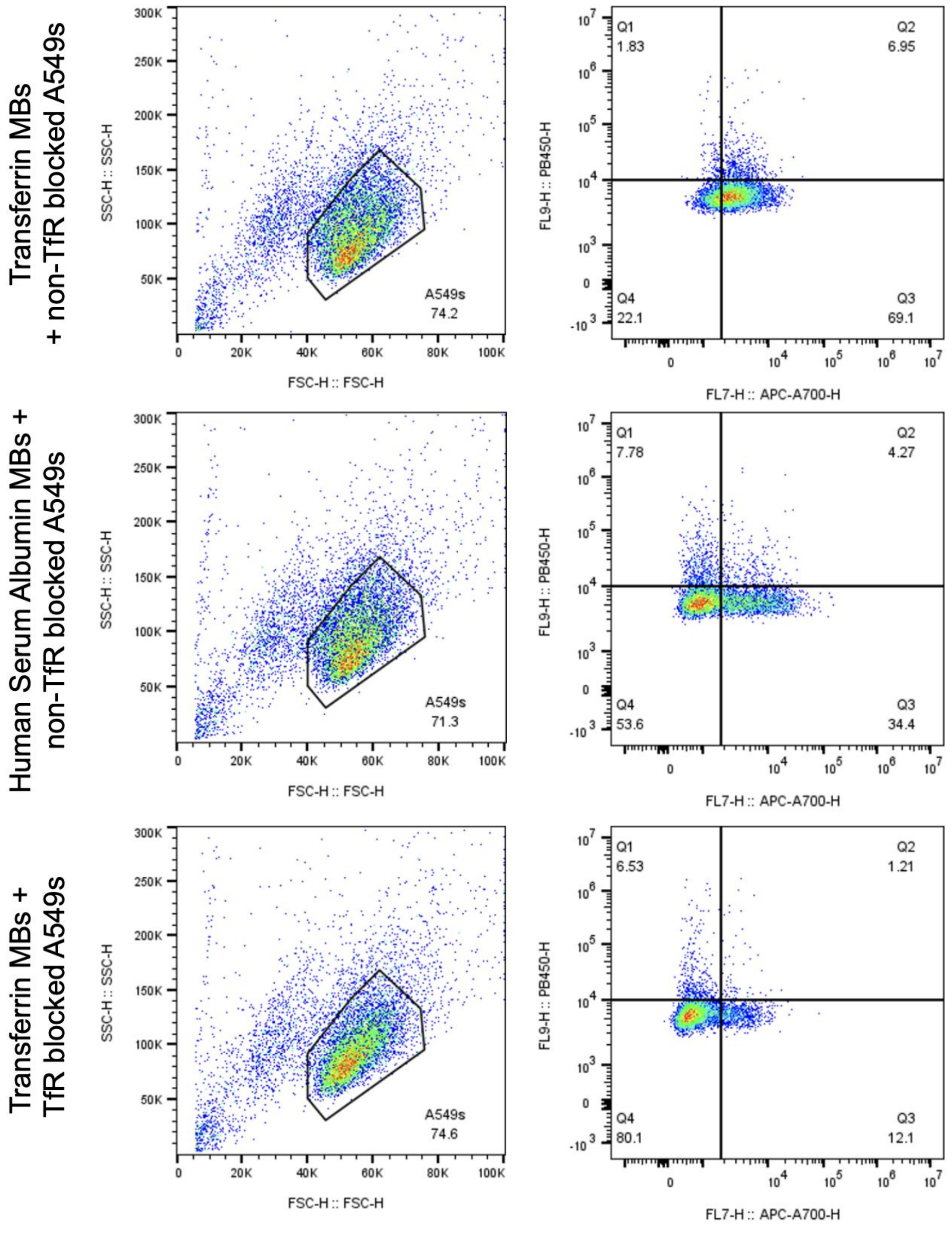

Figure S6. Examples of flow cytometry analysis of A549 cells treated with His-transferrin and His-human serum albumin microbubbles, at t = 35 minutes. Shown are the forward vs side scatter plots, and corresponding APC-A700 (CF^{TM}660C) vs PB450 (DAPI) plots for the A549 cells, corresponding to the live cell control, for non-TfR-blocked A549 cells plus both His-transferrin and His-human serum albumin microbubbles, and TfR-blocked A549 cells with His-transferrin microbubbles.

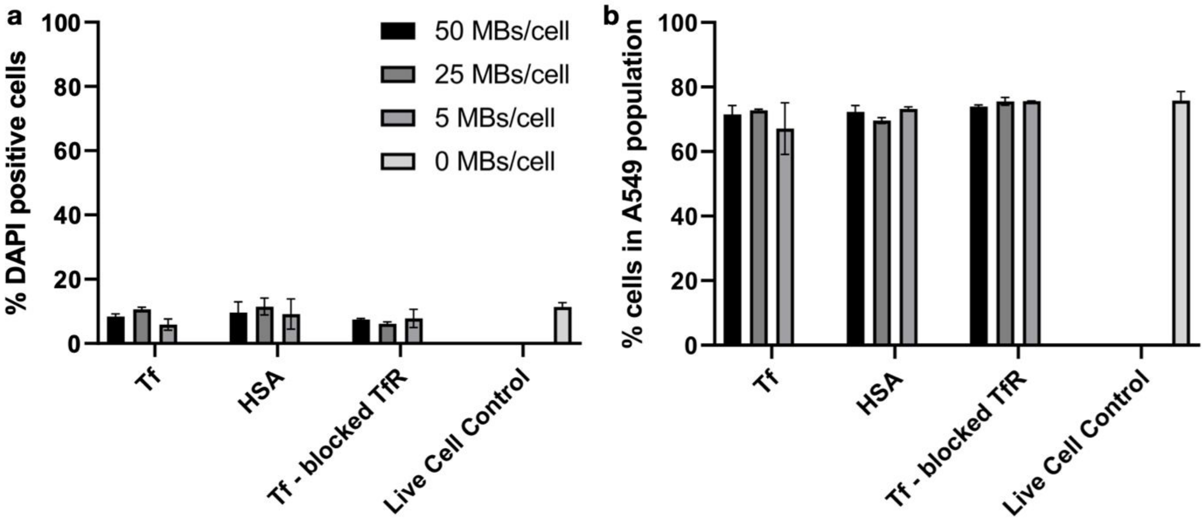

Figure S7. (a) Demonstration of cell death for each microbubble type (Tf- or HSA-loaded) and cell type (A549s with blocked and non-blocked TfR) at three microbubble concentrations (2, 5 and 10 x 10^7 MBs/mL, corresponding to 5, 25 and 50 MBs/cell) ($N = 3$ for Tf and $N = 2$ for HSA and Tf – blocked TfR), compared to the live cell control sample ($N = 4$). < 12% is DAPI positive and thus death. (b) To put this in perspective, the percentage of cells in the healthy population, the location in the FSC vs SSC diagram was identified based on the live cell control samples, is shown, demonstrating an insignificant effect of either microbubble type, concentration or TfR-blocking.

S8. Loading capacity of His-AF488-functionalised DGS-NTA(Ni) microbubbles

As stated in the experimental details section , DGS-NTA(Ni) microbubbles were incubated with 4 μg/mL of His-AF488. This section explores the theoretical maximum loading capacity and takes a preliminary look at the loading capacity as measured immediately after incubation of the microbubbles. There are two approaches to this calculation: 1) using the molar concentration of the nickelated lipid used in the microbubble formulation, and 2) using the total surface area that the His-tagged dye can occupy. The latter approach is taken as ICP-OES single-element spectroscopic analysis performed by Medac ltd revealed a fraction of nickelated lipids to be included in the microbubbles after bubble formulation.

A typical concentration of 3 x 10^9 MBs/mL and a typical surface mean diameter (D_{20} or D[2,0]) of 2 μm are used for this calculation. The surface mean diameter is the diameter of a hypothetical particle having a surface area equal to the mean surface area of the particles in a sample. This diameter can be calculated from the size distribution as:

$$D_{20} = \frac{\int_0^\infty D^2 p(D) dD}{\int_0^\infty p(D) dD} \tag{1}$$

, where *D* equals the particle diameter, and *p(D)* is the probability density function of the size distribution, used to calculate the total surface area[1]. Using the molecular footprint of a typical phospholipid or the His-tagged payload - whichever is larger, the total number of payload molecules that can fit on the microbubble surface area can be estimated. Using a typical phospholipid molecular area of 50 $Å^2$, or 5 x 10^{-19} m^2, and a total surface area of 3 x 10^9 $4\pi (D_{20}/2)^2$ which equals 0.04 m^2/mL, gives a total number of phospholipids equal to 2.4 μmol in 2 mL of microbubble suspension. If 2% of those are DGS- NTA(Ni), the upper bound on the amount of bound His-tagged AF488 is 6 μg.

Experimentally, the loading was measured immediately after the fabrication of the microbubbles. Using a fluorescence plate reader, a standard curve was obtained for His-AF488, as shown in **Figure S8**. After microbubble fabrication and centrifugation, the fluorescence of the microbubble-free subnatent was measured to obtain the concentration of unbound His-AF488. By subtracting this concentration from the total His-AF488 concentration of 4 µg/mL with which the microbubbles were incubated, the amount of His-AF488 present on the microbubble shell after fabrication and centrifugation can be estimated. This results in 2.1 ± 0.2 µg/mL remaining on the microbubbles immediately after fabrication (*N* = 3).

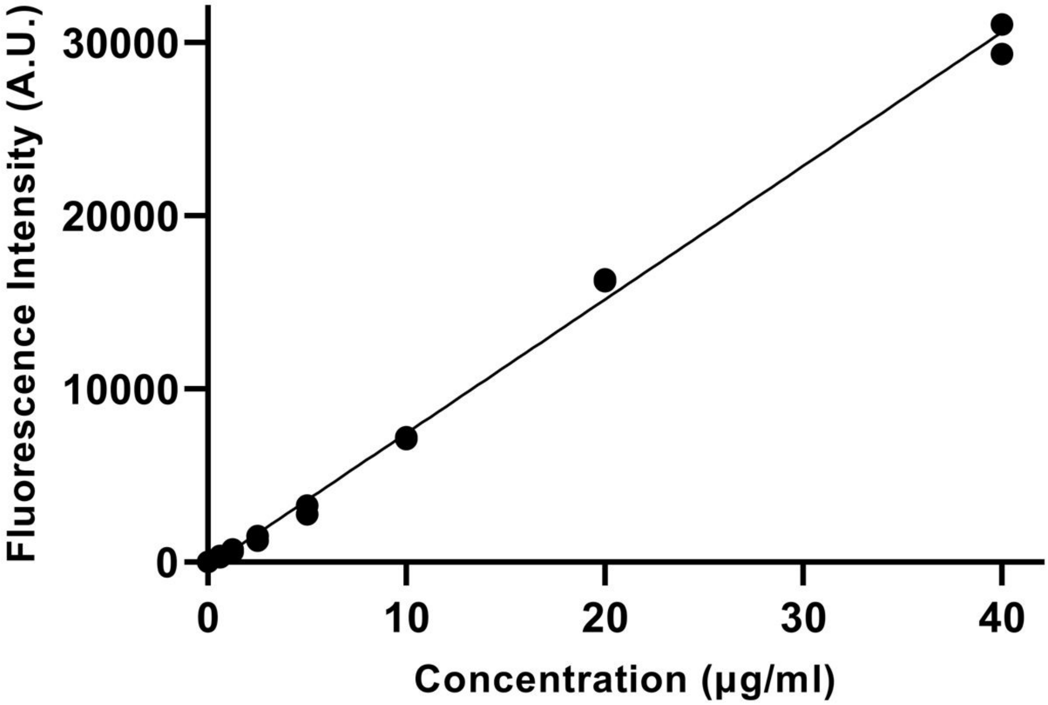

Figure S8. The standard curve for fluorescence of His-AF488. The data (N = 2) were obtained using a fluorescence plate reader (gain 600, excitation 490 ± 10 nm, emission 520 nm). The corresponding linear relationship is defined as y = 772.07x - 278.8 with an R^2 value of 0.9976.

Caution should be taken in interpreting this data, as the effect of resuspending the microbubbles prior to their use is not considered. The binding between mono-NTA(Ni) and a His_6-tag is

relatively weak, with a K_D of 74 13 ± 5 x 10^3 nM, and the dissociation kinetics fast, with a k_{off} of (1800 ± 400) x 10^{-3} s^{-1}, corresponding to a complex half-life of 0.39 seconds[2]. Consequently, when a microbubble cake containing only bound His-tagged molecules is resuspended, a new equilibrium will be established on a timescale of a few seconds, with a substantial decrease in the amount of bound His-tagged molecules on the microbubbles.

S9. ICP-OES single-element spectroscopic analysis

MEDAC LTD

Analytical and chemical consultancy services

ANALYTICAL REPORT ICP-OES

MEDAC Ltd
Alpha 319
Chobham Business Centre
Chertsey Road
Chobham
Surrey
GU24 8JB
United Kingdom

www.medacltd.com
Tel: 01276 855410
Email: info@medacltd.com

Date 23rd August 2023

Name Dr Veerle Brans

Company / Institution Oxford University

Sample Identification	Assay No.	Element	Results	Units
1	208480	Ni	468	ppm
3	208481	Ni	<10	ppm
4	208482	Ni	29	ppm

Comments

Analyst Stephen Goodall

Vers: 2022

Sample 1 is the lipid film prior to microbubble production and sample 3 and 4 are the freeze-dried samples of NTA(Ni)-functionalized microbubbles. The lipids in all three samples were resuspended in 2.2 mL of DPBS.

References

(1) Filippa, L.; Trento, A.; Álvarez, A. M. Sauter Mean Diameter Determination for the Fine Fraction of Suspended Sediments Using a LISST-25X Diffractometer. *Measurement (Lond)* **2012**, *45* (3), 364–368. https://doi.org/10.1016/j.measurement.2011.11.009.

(2) Lata, S.; Reichel, A.; Brock, R.; Tampé, R.; Piehler, J. High-Affinity Adaptors for Switchable Recognition of Histidine-Tagged Proteins. *J Am Chem Soc* **2005**, *127* (29), 10205–10215. https://doi.org/10.1021/JA050690C/SUPPL_FILE/JA050690CSI20050421_122054.PDF.
